# Comparative expression profiles of carboxylesterase orthologous *CXE14* in two closely related tea geometrid species, *Ectropis obliqua* Prout and *Ectropis grisescens* Warren

**DOI:** 10.3389/fphys.2023.1194997

**Published:** 2023-05-24

**Authors:** Fengshui Yang, Yujie Li, Mengyuan Gao, Qing Xia, Qian Wang, Meijun Tang, Xiaogui Zhou, Huawei Guo, Qiang Xiao, Liang Sun

**Affiliations:** ^1^ Key Laboratory of Tea Quality and Safety Control, Ministry of Agriculture and Rural Affairs, Tea Research Institute, Chinese Academy of Agricultural Sciences, Hangzhou, China; ^2^ Department of Plant Protection, Henan Institute of Science and Technology, Xinxiang, China; ^3^ College of Advanced Agricultural Sciences, Zhejiang A & F University, Hangzhou, China

**Keywords:** tea geometrid moth, sibling species, carboxylesterases, *Wolbachia*, expression profiles

## Abstract

Insect carboxylesterases (CXEs) can be expressed in multiple tissues and play crucial roles in detoxifying xenobiotic insecticides and degrading olfactory cues. Therefore, they have been considered as an important target for development of eco-friendly insect pest management strategies. Despite extensive investigation in most insect species, limited information on CXEs in sibling moth species is currently available. The *Ectropis obliqua* Prout and *Ectropis grisescens* Warren are two closely related tea geometrid species, which share the same host of tea plant but differ in geographical distribution, sex pheromone composition, and symbiotic bacteria abundance, providing an excellent mode species for studies of functional diversity of orthologous CXEs. In this study, we focused on *EoblCXE14* due to its previously reported non-chemosensory organs-biased expression. First, the *EoblCXE14* orthologous gene *EgriCXE14* was cloned and sequence characteristics analysis showed that they share a conserved motif and phylogenetic relationship. Quantitative real-time polymerase chain reaction (qRT-PCR) was then used to compare the expression profiles between two *Ectropis* spp. The results showed that *EoblCXE14* was predominately expressed in *E. obliqua* larvae, whereas *EgriCXE14* was abundant in *E. grisescens* at multiple developmental stages. Interestingly, both orthologous *CXEs* were highly expressed in larval midgut, but the expression level of *EoblCXE14* in *E. obliqua* midgut was significantly higher than that of *EgriCXE14* in *E. grisescens* midgut. In addition, the potential effect of symbiotic bacteria *Wolbachia* on the *CXE14* was examined. This study is the first to provide comparative expression profiles of orthologous *CXE* genes in two sibling geometrid moth species and the results will help further elucidate CXEs functions and identify a potential target for tea geometrid pest control.

## Introduction

Chemicals with ester functional groups such as species-specific pheromones, host plant odorants, and xenobiotic insecticides are important for insect physiology, biochemistry, and ecology (Xu and Turlings, 2018; Levi-Zada and Byers, 2021; Mdeni et al., 2022). Carboxylesterases (CXEs) belong to a superfamily that includes diverse functional enzymes (Oakeshott et al., 1999; Oakeshott et al., 2005). They hydrolyze esters to their corresponding acids and alcohols and are thus thought to be involved in xenobiotic detoxification, odorant degradation, and developmental regulations (Richmond et al., 1980; Leal, 2013; Godoy et al., 2021).

Insect CXEs possess highly conserved catalytic triads (Ser-Glu-His) and pentapeptide motifs (Gly-X-Ser-X-Gly). They are expressed in the midgut, fat body, and other detoxifying organs which metabolize pesticide organophosphates, pyrethroids, and carbamates in various insect species such as moths *Helicoverpa armigera* (Bai et al., 2019), *Grapholita molesta* (Li et al., 2023), *Plutella xylostella* (L.) (Li et al., 2021), and *Spodoptera frugiperda* (Carvalho et al., 2013); the aphid *Aphis gossypii* (Chang et al., 2010); and flies *Lucilia cuprina* (Heidari et al., 2005) and *Drosophila melanogaster* (Birner-Gruenberger et al., 2012). The overexpression of *CXE* genes has been reported as an important mechanism of insecticide detoxification. For instance, overexpressed *CXE* genes have been shown to be involved in indoxacarb resistance in *Spodoptera litura* (Shi et al., 2022); an overexpressed CXE (PxαE14) in the detoxification of multiple insecticides in *P. xylostella* (Li et al., 2022); and two overexpressed a-esterase genes mediated metabolic resistance to malathion in the oriental fruit fly, *Bactrocera dorsalis* (Hendel) (Wang et al., 2015). Additionally, the alternative splicing and site mutation of CXEs are considered another mechanism in insecticide detoxification. The alternative splicing of TcCCE23 with different variants enhances fenpropathrin tolerance in *Tetranychus cinnabarinus* (Wei et al., 2023). Two different amino acid substitutions in the ali-esterase E3 confer alternative types of organophosphorus insecticide resistance in the sheep blowfly, *Lucilia cuprin* (Campbell et al., 1998).

In addition to xenobiotic detoxification, some CXEs have been demonstrated to be highly expressed at the antennae, the main insect olfactory organs, and function as odorant degrading enzymes (ODEs) that play crucial roles in olfactory cue degradation. The first insect ODE (ApolPDE) was identified from the silk moth *Antheraea polyphemus*; it displayed male antennae-specific expression and could rapidly degrade *A. polyphemus* major acetate sex pheromone, (*E*, Z)-6, 11-hexadecadienyl acetate [E6Z11–16Ac] (Vogt and Riddiford, 1981; Ishida and Leal, 2005). Another male antennae-specific *CXE* gene, the encoded protein (PjapPDE) of which was identified in the Japanese beetle, *Popillia japonica,* could degrade the sex pheromone component (R, Z)-5-(-)-(1-decenyl) oxacyclopentan-2-one [(R)-japonilure] (Ishida and Leal, 2008). In addition, an extracellular CXE, named esterase-6 (Est-6), in *D. melanogaster* was reported to be highly expressed in olfactory sensilla and involved in maintaining proper temporal dynamics of *cis*-vaccenyl acetate (cVA) detection (Chertemps et al., 2012). Subsequently, an increasing number of insect CXEs have been identified and functionally reportedly to be involved in degradation of both plant odorants and sex pheromones (Durand et al., 2010a; Durand et al., 2010b; Durand et al., 2011; Zhang et al., 2016; He et al., 2020).

*Ectropis obliqua* Prout and *Ectropis grisescens* Warren are two closely related tea geometrid species which have frequent outbreaks in tea gardens and result in serious damages to tea production (Zhang et al., 2014). At present, chemical control is still the main method against these two sibling species, which accelerates the risk of resistance development. A better understanding of the mechanism of insecticide detoxification and screening of the target genes involved in xenobiotic detoxification and odorant degradation is crucial for development of environmental friendly pest management strategies. The CXEs present potential targets due to their important roles in detoxifying various insecticides and degrading olfactory cues.

Previously, 35 *EoblCXE* genes have been identified in *E. obliqua* chemosensory organs and they displayed multiple tissues expression profiles (Sun et al., 2017). Whether these EoblCXEs in *E. obliqua* have their orthologs in the sibling species *E. grisescens* and the differences in their expression profiles are still unknown. *Ectropis obliqua* differs from *E. grisescens* in geographical distribution, sex pheromone composition, and symbiotic bacteria *Wolbachia* abundance (Luo et al., 2017; Li et al., 2019; Wang et al., 2020). Whether the differences in *Wolbachia* abundance are associated with CXE expression patterns should be investigated.

In this study, we focused on *EoblCXE14*, which showed a non-chemosensory organs-biased expression at the abdomen of *E. obliqua* adults (Sun et al., 2017). First, we cloned its orthologous gene *EgriCXE14* in *E. grisescens* and analyzed its sequence characteristics; then we compared the expression profiles between the sibling species *E. obliqua* and *E. grisescens* and evaluated the potential effect of *Wolbachia* on their expression pattern. The results will help further investigation of the potential roles of *CXE14* in xenobiotic detoxification.

## Materials and methods

### Insect rearing

The larvae of *E. obliqua* and *E. grisescens* was collected from Yuhang and Yueqing tea plantations in Zhejiang Province, respectively and have been reared for multiple generations in the laboratory. Identification of species to distinguish *E. obliqua* and *E. grisescens* was performed according to previous studies (Zhang et al., 2014). Larvae were fed on the fresh tea cultivar and reared under 25°C ± 1°C with 65% ± 5% humidity and L13:D11 photoperiod. The male and female pupae were identified and separated for eclosion.

The mutant population of *E. grisescens,* which was collected from Yizheng, Jiangsu province, and its *Wolbachia* was removed by using tetracycline, has been previously established and reared in our laboratory for many generations. PCR detection of the *wsp* gene was performed as described in previous reports (Baldo et al., 2006; Zhou et al., 2016) to ensure the removal of *Wolbachia* in the *E. grisescens* mutant with the *COI* gene for checking the quality of extracted DNA templates as described by Zhang et al. (2014). The rearing conditions of *E. grisescens* mutant were the same as those of *E. obliqua* and *E. grisescens* described above.

### Tissue collection

For the developmental expression pattern analysis of *CXE14* in *E. obliqua* and *E. grisescens*, four biological replicates were collected, and each biological replicates contained the following: 200 eggs (day 0), 15 third instar larvae, 5 pupae (day 5–6), and 5 new emergence moths (day 0) of each sex. For the tissue-specific expression patterns of *CXE14*, 200 third instar larvae of *E. obliqua, E. grisescens,* and *E. grisescens* mutant were dissected into head, midgut, fat body, and epidermis, respectively. Four replicates were collected for each tissue sample. All of the specimens were collected and immediately stored in −80°C until use.

### RNA extraction and cDNA synthesis

Total RNA was extracted using the Trizol reagent (Thermo, United States) according to the manufacturer’s protocol. Purity was assessed using NanoDrop2000 (Thermo, United States) and the quality of total RNA was detected in 1.0% agarose electrophoresis. First-strand cDNA was synthesized from 1 µg RNA using a MonScript™ RTⅢ Super Mix with dsDNase (Two-Step) (Monad, Wuhan, China).

### *EgriCXE14* gene clone

Homology-based cloning was used to clone *EgriCXE14* in *E. grisescens*; the specific primers (Supplementary Table S1) were designed based on its ortholog *EoblCXE14* (Genbank accession No. KX015856.1) (Sun et al., 2017). PCR was performed using a MonAmp™ 2 x Taq Mix Pro (+Dye) (Monad, Wuhan, China) under the following condition: 94°C for 3 min, followed by 40 cycles at 95°C for 30 s, 65°C for 30 s, 72°C for 2 min, and a final elongation step at 72°C for 10 min. The 50 μL reaction mixture contained 25 μL MonAmpTM 2 × Taq Mix Pro(+Dye), 2 μL of each forward and reverse primers, 2 μL cDNA template, 19 μL ddH2O. The PCR product was analyzed in 1.5% agarose electrophoresis.

The PCR products were purified using a DNA Gel Extraction Kit (Axygen, Shanghai, China), and connected to pCE2 TA/Blunt-Zero vector (Vazyme, Nanjing, China) with a 5 μL reaction mixture containing 1 μL 5× TA/Blunt-Zero Cloning Mix and 4 μL purified DNA product. The product was incubated at 37°C for 30 min and then transferred into *Escherichia coli* DH5α (AngYuBio, Shanghai, China). Positive colonies were selected using PCR using specific primers for sequencing.

### Sequence alignment and phylogenetic analysis

The amino acid sequences of EgriCXE14 and previously functional reported HarmCarE001a, HarmCarE001g, BmorCarE, and LcaE7 from *H. armigera* (Bai et al., 2019), *Bombyx mori* (Cui et al., 2011), and *L. cuprina* (Jackson et al., 2013), respectively, were aligned using ClustalW (https://www.genome.jp/tools-bin/clustalw). MEME web service was used to identify conserved motifs (https://meme-suite.org/meme/index.html). The SMART website was selected to characterize the domain in targeted protein sequences (http://smart.embl-heidelberg.de/). A neighbor-joining tree was constructed using MEGA11.0 software with p-distance amino acid substitution (Tamura et al., 2021). Bootstrapping with 1,000 replicates was used to evaluate the reliability of the tree topology. The protein accession numbers of CXEs used in phylogenetic tree construction are listed in Supplementary Table S2.

### Quantitative real-time polymerase chain reaction (qRT-PCR)

qRT-PCR was performed using Roche Light Cycle 480 (Roche, Swiss) and the specific primers of *CXE14* were designed using Primer Premier 5.0 (Supplementary Table S1). The glyceraldehyde-3-phosphate dehydrogenase *EoblGAPDH* (Genbank accession No. KT991373) and its specific primers previously used for qPCR (*EoblGAPDH*-F: TAT​CTC​TCT​GAA​CGA​CAA​CTT; *EoblGAPDH*-R: TTG​GTC​TGG​ATG​TAC​TTG​AT) (Yan et al., 2020) were used to normalize the target gene *CXE14* expression and correct the sample-to-sample variation. Each reaction was conducted in a 10 μL reaction mixture containing 5 μL TB Green Premix Ex Taq II, 0.4 μL of each primer, 1 μL cDNA template (approximately 200 ng), and 3.2 μL sterilized H_2_O. The qRT-PCR parameters were as follows: 95°C for 30 s, followed by 40 cycles at 95 °C for 5 s, and 60°C for 30 s. Then, fluorescence was measured using a 55°C–95°C melting curve to detect a specific-peak for individual gene. Each reaction was performed in three technical replicates and four independent biological replicates.

The relative fold gene expression levels were calculated using the 2^−ΔΔCT^ method (Livak and Schmittgen, 2001), and the statistical significance of the differences was analyzed using SPSS Statistics 26.0 software. The comparison of the relative expression levels of *CXE14* amongst the developmental stages and tissues of *E. obliqua* and *E. grisescens* was conducted using a one-way nested analysis of variance (ANOVA), followed by the least significant difference test (LSD) at α = 0.05. The relative expression levels of *CXE14* between two samples were determined using an independent-samples *t*-test. GraphPad Prism 9 software was used to create graphs.

## Results

### Identification and sequence characteristics of CXE14

**FIGURE 1:**
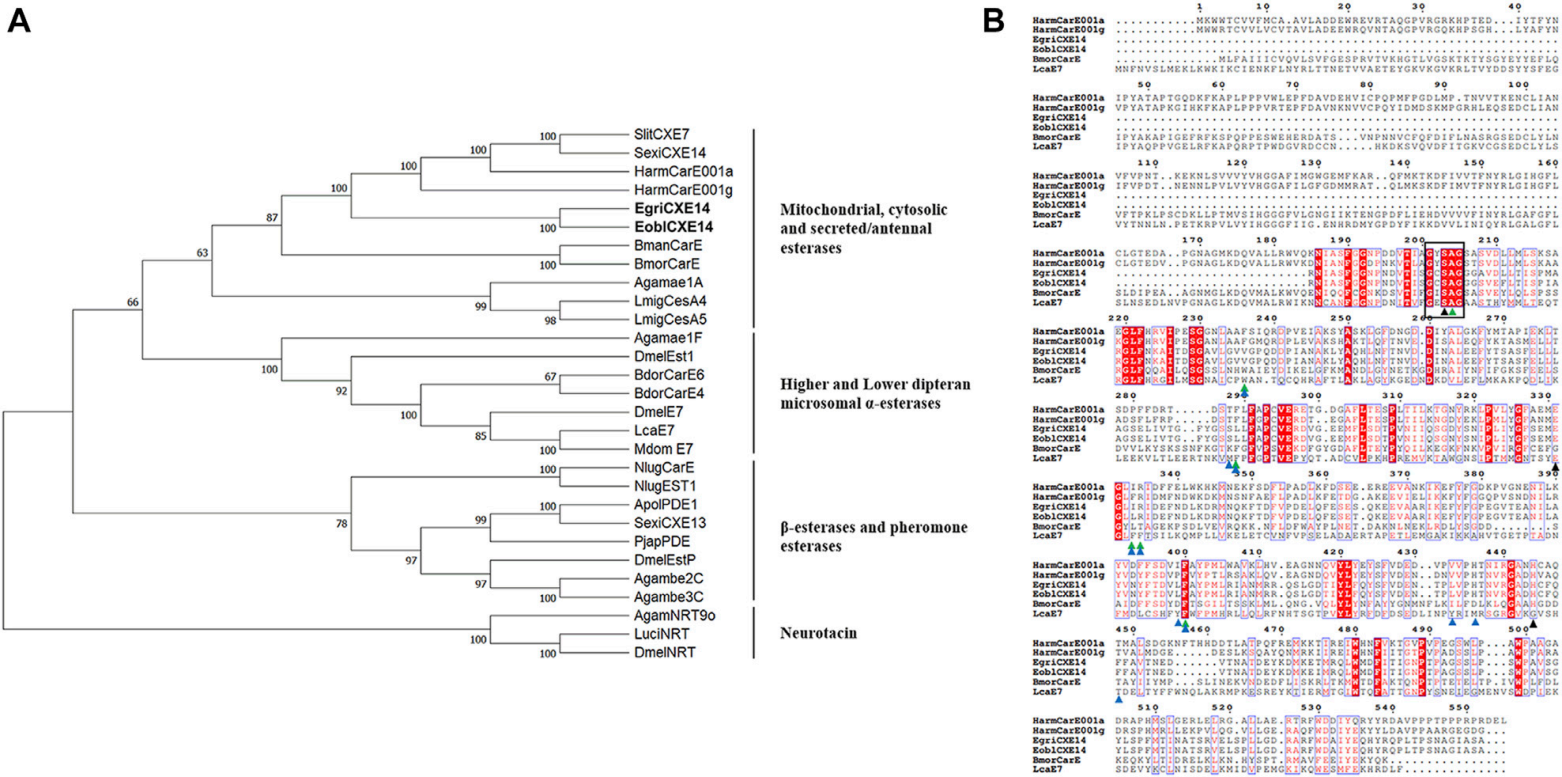
**(A)** Phylogenetic analyses of CXE14. CXE sequences used in this analysis are shown in Supplementary Table S2. EoblCXE14 and EgriCXE14 are shown in bold. **(B)** Sequence characteristics of EoblCXE14 and EgriCXE14 with previously functional reported HarmCarE001a, HarmCarE001g, BmorCarE, and LcaE7. The catalytic triad residues (Ser-His-Glu) are marked with a black triangle, the highly conserved pentapeptide residues (GXSXA) are marked with a black box; the location of key amino acids corresponding to binding pocket residues of LcaE7 and HarmCarE001g are marked with blue and green triangles, respectively.

The sequence of EgriCXE14 was identified from the adult head of *E. grisescens* using a homologous cloning method. The obtained partial-length sequence of EgriCXE14 was 1,098 bp and encoded 366 amino acids; it was deposited in GenBank (accession No. OQ701617). The sequence identity between orthologs EgriCXE14 and EoblCXE14 was 97.8%, and amino acid residue variations were found at Ala24Ser, Asp26Glu, Leu27Phe, Met33Ile, Asp136Asn, Ile153Leu, Ser183Thr, and Thr288Ile (Supplementary Figure S1). The conserved motif of EgriCXE14 and EoblCXE14 was analyzed and the results showed that two CXE14 orthologs had insect CXE conserved functional domains such as the catalytic triads (Ser-His-Glu) and pentapeptide motif (GXSXA) (Figure 1).

### Phylogenetic analyses of CXE14

To deduce potential roles of CXE14, the phylogenetic relationships of EgriCXE14 and EoblCXE14 with some carboxylesterases which have been previously functional reported in insecticide detoxification were analyzed. The results showed that EgriCXE14 and EoblCXE14 were clustered into the HarmCarE001g clade, a carboxylesterase reportedly associated with pyrethroids detoxification in *H. armigera* (Figure 1). In addition, the SMART analysis showed that EgriCXE14 and EoblCXE14 contained similar a COesterase to HarmCarE001g and HarmCarE001a (Supplementary Figure S2).

### Developmental expression profiles of *CXE14* in *E. obliqua* and *E. grisescens*

**FIGURE 2:**
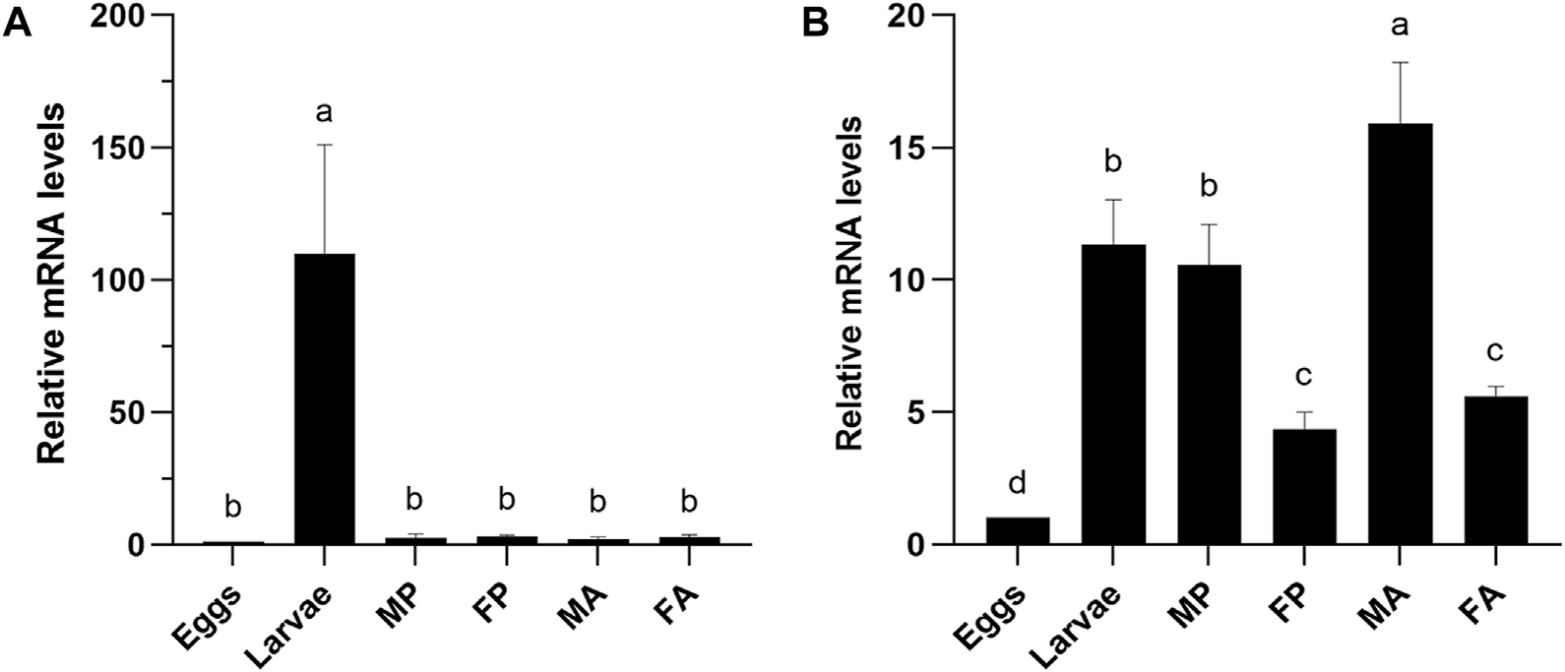
The expression profiles of *CXE14* at different developmental stages of *E. obliqua* **(A)** and *E. grisescens* **(B)**. MP, male pupae; FP, female pupae; MA, male adults; FA, female adults; The error bars represent the standard deviation (SD), different letters above each bar denote significant differences (one-way ANOVA followed by LSD test, *p* < 0.05).

The expression profile of orthologous gene *CXE14* in the two sibling species *E. obliqua* and *E. grisescens* at different developmental stages, including eggs, larvae, male and female pupae, male and female adults, were examined using qRT-PCR. The results showed that the two *CXE14* orthologs were detected at all the examined developmental stages but exhibited a notably different expression pattern. In *E. obliqua*, *EoblCXE14* was predominately expressed in larvae (Figure 2A). By contrast, *EgriCXE14* was highly abundant in male adults, followed by larvae, male pupae, female adults, female pupae, and eggs (Figure 2B).

### Tissue-specific expression patterns of *CXE14* in *E. obliqua* and *E. grisescens* larvae

**FIGURE 3:**
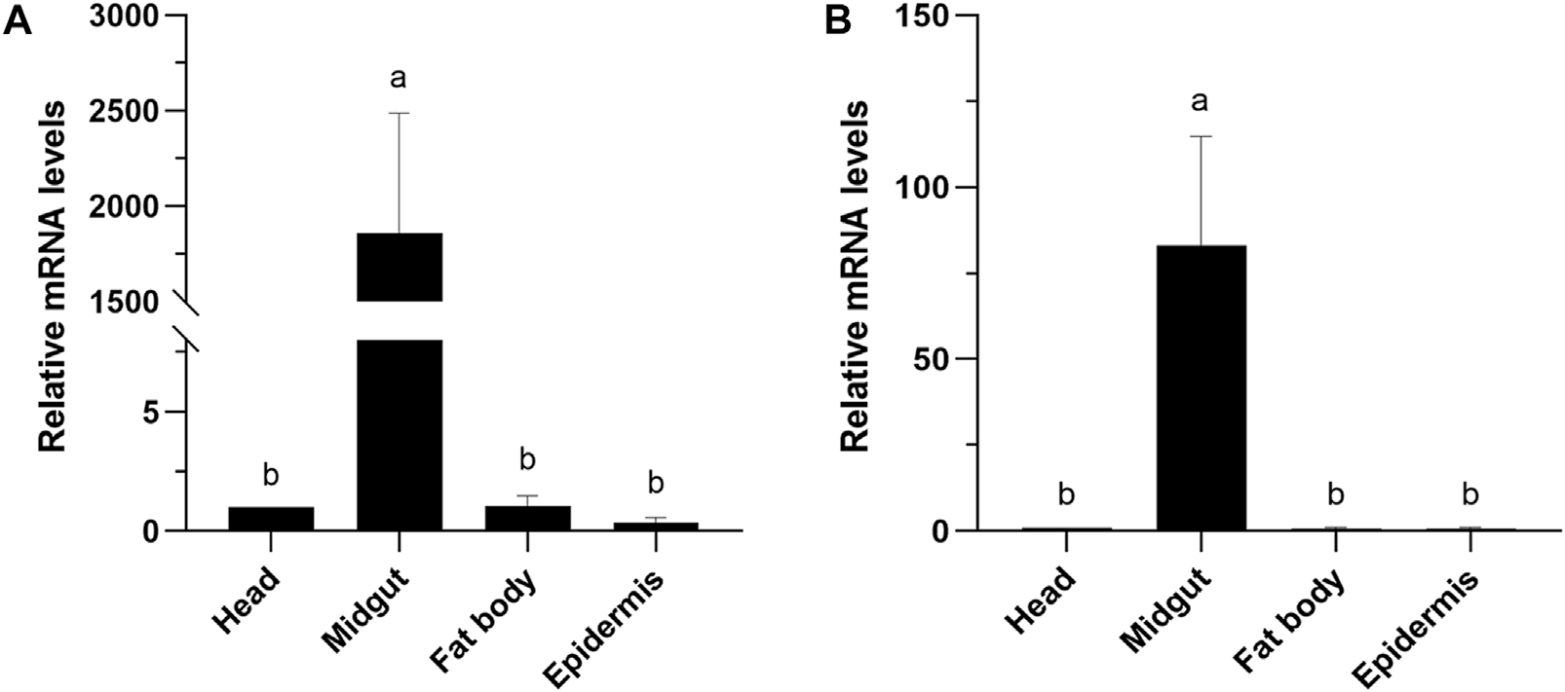
Tissue-specific expression patterns of *CXE14* in *E. obliqua* **(A)** and *E. grisescens* **(B)** larvae. The error bars represent the standard deviation (SD); different letters above each bar denote significant differences (one-way ANOVA followed by LSD test, *p* < 0.05).

The expression levels of *CXE14* orthologs in different tissues of *E. obliqua* and *E. grisescens* larvae were studied. The qRT-PCR results showed that the two orthologs *EoblCXE14* and *EgriCXE14* had a conserved tissue-specific expression pattern; the expression levels of *EoblCXE14* and *EgriCXE14* were significant higher in the midgut than those in the heads, fat body, and epidermis (Figure 3).

### Comparative expression of *CXE14* between *E. obliqua* and *E. grisescens*

**FIGURE 4:**
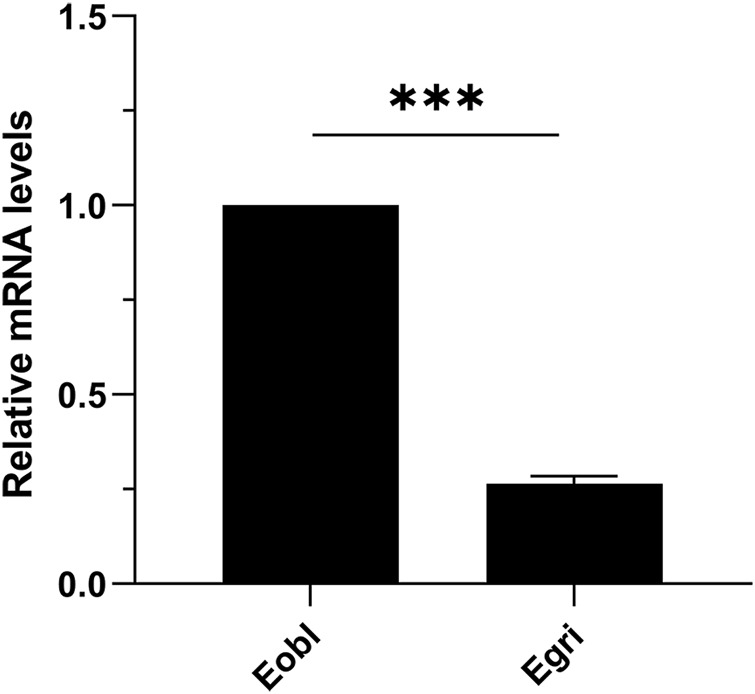
The comparison of *CXE14* expression levels in the midgut of *E. obliqua* and *E. grisescens*. Eobl, *E. obliqua*; Egri, *E. grisescens*; the error bars represent the standard deviation (SD), *** indicates significant differences in *CXE14* expression between *E. obliqua* and *E. grisescens* (independent-samples *t*-test, *p* < 0.001).

To further elucidate the functional differences of *CXE14* orthologs in sibling species, we compared the expression levels of *EoblCXE14* and *EgriCXE14* in the midgut of *E. obliqua* and *E. grisescens* using qRT-PCR. The results showed that the expression level of *EoblCXE14* in the *E. obliqua* midgut was higher (approximately 3.8-fold) than that of *EgriCXE14* in the *E. grisescens* midgut (Figure 4).

### Potential effect of *Wolbachia* on the *CXE14* expression

The mutant population of *E. grisescens* with *Wolbachia* removed using tetracycline has been previously constructed in our laboratory. PCR specific for the *wsp* gene was performed and the results showed a clear *wsp* gene band in *E. grisescens*, but not in the *E. grisescens* mutant, indicating a successful removal of *Wolbachia* from *E. grisescens* larvae (Supplementary Figure S3).

**FIGURE 5:**
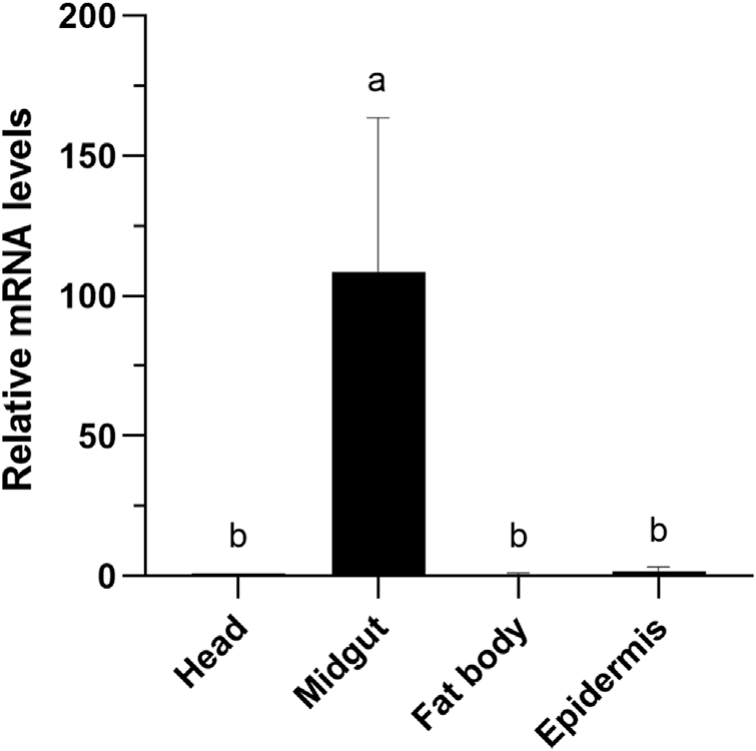
Tissue-specific expression patterns of *CXE14* in *E. grisescens* larvae mutant. The error bars represent the standard deviation (SD); different letters above each bar denote significant differences (one-way ANOVA followed by LSD test, *p* < 0.05).

Tissue-specific expression pattern of *EgriCXE14* in the *E. grisescens* larval mutant was examined using qRT-PCR. The results showed that *EgriCXE14* in the *E. grisescens* mutant was highly expressed in the midgut (Figure 5).

**FIGURE 6:**
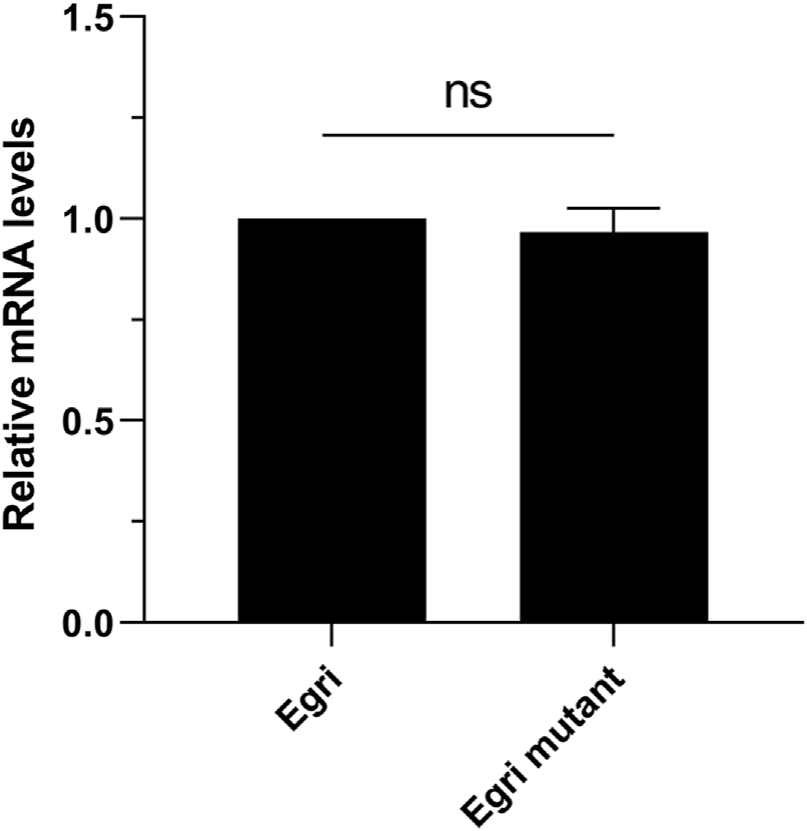
The potential effect of *Wolbachia* on the expression level of *CXE14* in the midgut of *E. grisescens*. Egri, *E. grisescens*; Egri mutant, *E. grisescens* without *Wolbachia*; The error bars represent the standard deviation (SD), ns indicates no significant difference in *CXE14* expression level between *E. grisescens* and *E. grisescens* mutant (independent-samples *t*-test, *p* > 0.05).

To deduce the potential effect of *Wolbachia* on the expression difference of *EgriCXE14*, we compared the expression levels of *EgriCXE14* in larval midgut between *E. grisescens* and *E. grisescens* mutant. The qRT-PCR results showed that the expression level of *EgriCXE14* in *E. grisescens* mutant was not significantly different from that in *E. grisescens* (Figure 6).

## Discussion

Insect CXEs have been widely demonstrated to be involved in pesticide detoxification and pheromone and plant odorant degradation. Therefore, they represent a major target for development of eco-friendly pest management strategies. This study reports a pair of *CXE* orthologous genes, *EoblCXE14* and *EgriCXE14*, which share a conserved motif and phylogenetic relationship but have different expression profiles in two closely related tea geometrid species, *E. obliqua* and *E. grisescens*.

Insect CXEs have been identified in most of the lepidopteran moth species, such as *Spodoptera littoralis* (Durand et al., 2010b), *S. litura* (Zhang et al., 2016), *Carposina sasakii* (Li and Zhang, 2022), *Hyphantria cunea* (Ye et al., 2021), and *P. xylostella* (Xie et al., 2017; Li et al., 2021; Wang et al., 2021), which represent important pests of agricultural food crops, cotton, fruit trees, and vegetables. By contrast, the identification of CXEs in tea plant pest, especially in the sibling species *E. obliqua* and *E. grisescens,* have not been well explored, although we have previously identified and characterized 35 CXEs in tea geometrid moth *E. obliqua* (Sun et al., 2017). In this study, we cloned a *CXE* gene from *E. grisescens* named *EgriCXE14* which is the orthologs of *EoblCXE14*. Although it was not the full-length sequence, *EgriCXE14* contained the main functional domain of insect CXEs including the highly conserved pentapeptide residues (GXSXA) and the Ser-His-Glu catalytic triad residues. In addition, some amino acid residues such as Arg, Phe, and His, which are considered key residues in the binding pocket associated with insecticide binding of *H. armigera* HarmCarE001g (Bai et al., 2019) or *L. cuprina* LcaE7 (Jackson et al., 2013), are also conserved in both EoblCXE14 and EgriCXE14 (Figure 1), indicating potential roles of these two *CXE* genes in detoxifying insecticides.

The phylogenetic analyses and larval tissue-specific expression patterns observed in this study support the above-mentioned speculation. EgriCXE14 and EoblCXE14 were found to cluster with HarmCarE001g, a carboxylesterase which has been demonstrated to detoxify pyrethroids in *H. armigera* (Bai et al., 2019) (Figure 1). Furthermore, qRT-PCR results showed that both *EgriCXE14* and *EoblCXE14* were highly expressed in the midgut, a detoxification metabolic organ of *E. obliqua* and *E. grisescens* larvae (Figure 3). Indeed, many CXEs of different moth species are highly detected in the midgut and play crucial roles in insecticide detoxification. The *CXEs* genes (CarE) in *G. molesta* have higher expression in the detoxification metabolic organs such as the midgut and fat body but lower expression in the head and cuticle, and are involved in the tolerance of insecticides (Li et al., 2023). In the diamondback moth, *P. xylostella*, PxEst-6 is highly expressed in the midgut and cuticles of the third instar larvae and is associated with pyrethroid insecticides metabolization (Li et al., 2021), whereas PxαE14 is predominantly expressed in the midgut and Malpighian tubule of larvae and contributes to detoxification of multiple insecticides (Li et al., 2022). *CXE* genes (*SlituCOE*) of *S. litura* are expressed in the midgut, Malpighian tubule, and fat body, and are related to insecticide indoxacarb resistance (Shi et al., 2022). Further functional studies of enzyme activities and RNAi knockdown are needed to test this hypothesis in two closely related tea geometrid *Ectropis* spp.

Despite the predominant expression in the larval midgut, the expression profiles of *EoblCXE14* and *EgriCXE14* are significantly different. The investigation of expression profiles at different developmental stages showed that *EoblCXE14* was larvae-enriched in *E. obliqua* but *EgriCXE14* was ubiquitously expressed at different developmental stages of *E. grisescens* (Figure 2). These results indicate that *EoblCXE14* likely plays roles in *E. obliqua* larvae; however, *EgriCXE14* probably functions in *E. grisescens* at multiple developmental stages. A possible role of *CXE14* in degrading ester odorants cannot be excluded, because several CXEs in insect species reportedly contribute to degradation of insect pheromones and plant volatiles (Ishida and Leal, 2005; Durand et al., 2011; Chertemps et al., 2012; He et al., 2020).

The expressional comparison in the larval midgut of the two *Ectropis* spp. showed that the expression level of *EoblCXE14* was significant higher than that of *EgriCXE14* (Figure 4). Previous studies proposed that *E. obliqua* differs from *E. grisescens* in symbiotic bacteria composition, particularly the *Wolbachia* (Zhou et al., 2016; Wang et al., 2020); specifically, *E. grisescens* has a significant higher abundance of *Wolbachia* than *E. obliqua*. *Wolbachia* has been shown to affect the expression of genes in insect (Pfarr et al., 2008; Cai et al., 2021; Sun et al., 2022). Therefore, in this study, we tested whether *Wolbachia* was correlated with different expression levels of *CXE14* in the midgut of the two *Ectropis* spp. The qRT-PCR results showed that *EgriCXE14* in *E. grisescens* mutant, in which *Wolbachia* was removed using tetracycline, was also highly expressed in the midgut (Figure 5), and no significant differences in *EgriCXE14* expression levels between *E. grisescens* and *E. grisescens* mutant were found (Figure 6). These results suggest that *Wolbachia* may not be related to *EgriCXE14* expression in the *E. grisescens* midgut, and may not contribute to the different expression level in the midgut of these two sibling species. These findings are not consistent with our previous reports that *Wolbachia* could be correlated with the expression of a chemosensory gene *CSP8* in *E. obliqua* and *E. grisescens* (Yan et al., 2022). Notably, *E. grisescens* mutant collected from the Yizheng, Jiangsu province, differs from the *E. grisescens* from Yueqing, Zhejiang province; therefore, we cannot rule out the possible effect of geographical population on the aforementioned findings. Further expression variation of target genes in different geographical population, and between wild (with *Wolbachia*) and mutant (without *Wolbachia*) *E. grisescens* from the same geographical region need to be evaluated.

In conclusion, this study characterized a pair of CXEs orthologs, *EoblCXE14* and *EgriCXE14,* in two sibling geometrid mode species, *E. obliqua* and *E. grisescens*. The results revealed that *EoblCXE14* and *EgriCXE14* share a conserved motif and phylogenetic relationship but differ in expression profiles at different developmental stages and expression levels in the larval midgut. *EoblCXE14* was predominately expressed in the *E. obliqua* larvae, whereas *EgriCXE14* was abundant in *E. grisescens* at multiple developmental stages. The expression level of *EoblCXE14* in the *E. obliqua* midgut was significantly higher than that of *EgriCXE14* in the *E. grisescens* midgut. In addition, potential effects of *Wolbachia* on the *CXE14* expression levels were examined. This study provides the first report on comparative expression profiles of orthologous *CXE* genes in two sibling geometrid moth species and could help identify potential targets for controlling tea geometrid.

## Data Availability

The datasets presented in this study can be found in online repositories. The names of the repository/repositories and accession number(s) can be found in the article/Supplementary Material.

## References

[B1] Bai L.-S. Zhao C.-X. Xu J.-J. Feng C. Li Y.-Q. Dong Y.-L. (2019). Identification and biochemical characterization of carboxylesterase 001G associated with insecticide detoxification in *Helicoverpa armigera* . Pestic. Biochem. Phys. 157, 69–79. 10.1016/j.pestbp.2019.03.009 31153479

[B2] Baldo L. Dunning Hotopp J. C. Jolley K. A. Bordenstein S. R. Biber S. A. Choudhury R. R. (2006). Multilocus sequence typing system for the endosymbiont *Wolbachia* pipientis. Appl. Environ. Microbiol. 72 (11), 7098–7110. 10.1128/aem.00731-06 16936055 PMC1636189

[B3] Birner-Gruenberger R. Bickmeyer I. Lange J. Hehlert P. Hermetter A. Kollroser M. (2012). Functional fat body proteomics and gene targeting reveal *in vivo* functions of *Drosophila melanogaster* α-Esterase-7. Insect Biochem. Mol. Biol. 42 (3), 220–229. 10.1016/j.ibmb.2011.12.004 22198472

[B4] Cai T. Zhang Y. Liu Y. Deng X. He S. Li J. (2021). *Wolbachia* enhances expression of NlCYP4CE1 in *Nilaparvata lugens* in response to imidacloprid stress. Insect Sci. 28 (2), 355–362. 10.1111/1744-7917.12834 32519810

[B5] Campbell P. M. Newcomb R. D. Russell R. J. Oakeshott J. G. (1998). Two different amino acid substitutions in the ali-esterase, E3, confer alternative types of organophosphorus insecticide resistance in the sheep blowfly, *Lucilia cuprina* . Insect Biochem. Mol. Biol. 28 (3), 139–150. 10.1016/s0965-1748(97)00109-4

[B6] Carvalho R. A. Omoto C. Field L. M. Williamson M. S. Bass C. (2013). Investigating the molecular mechanisms of organophosphate and pyrethroid resistance in the fall armyworm *Spodoptera frugiperda* . PLoS One 8 (4), e62268. 10.1371/journal.pone.0062268 23614047 PMC3629120

[B7] Chang J. Cao C. W. Gao X. W. (2010). The effect of pretreatment with S,S,S-tributyl phosphorotrithioate on deltamethrin resistance and carboxylesterase activity in *Aphis gossypii* (Glover) (Homoptera: Aphididae). Pestic. Biochem. Phys. 98 (2), 296–299. 10.1016/j.pestbp.2010.06.021

[B8] Chertemps T. François A. Durand N. Rosell G. Dekker T. Lucas P. (2012). A carboxylesterase, Esterase-6, modulates sensory physiological and behavioral response dynamics to pheromone in *Drosophila* . BMC Biol. 10, 56. 10.1186/1741-7007-10-56 22715942 PMC3414785

[B9] Cui F. Lin Z. Wang H. Liu S. Chang H. Reeck G. (2011). Two single mutations commonly cause qualitative change of nonspecific carboxylesterases in insects. Insect Biochem. Mol. Biol. 41 (1), 1–8. 10.1016/j.ibmb.2010.09.004 20888910

[B10] Durand N. Carot-Sans G. Bozzolan F. Rosell G. Siaussat D. Debernard S. (2011). Degradation of pheromone and plant volatile components by a same odorant-degrading enzyme in the cotton leafworm, *Spodoptera littoralis* . PLoS One 6 (12), e29147. 10.1371/journal.pone.0029147 22216190 PMC3246455

[B11] Durand N. Carot-Sans G. Chertemps T. Bozzolan F. Party V. Renou M. (2010a). Characterization of an antennal carboxylesterase from the pest moth *Spodoptera littoralis* degrading a host plant odorant. PLoS One 5 (11), e15026. 10.1371/journal.pone.0015026 21124773 PMC2993938

[B12] Durand N. Carot-Sans G. Chertemps T. Montagné N. Jacquin-Joly E. Debernard S. (2010b). A diversity of putative carboxylesterases are expressed in the antennae of the noctuid moth *Spodoptera littoralis* . Insect Mol. Biol. 19 (1), 87–97. 10.1111/j.1365-2583.2009.00939.x 20002215

[B13] Godoy R. Machuca J. Venthur H. Quiroz A. Mutis A. (2021). An overview of antennal esterases in Lepidoptera. Front. Physiol. 12, 643281. 10.3389/fphys.2021.643281 33868009 PMC8044547

[B14] He P. Mang D.-Z. Wang H. Wang M.-M. Ma Y.-F. Wang J. (2020). Molecular characterization and functional analysis of a novel candidate of cuticle carboxylesterase in *Spodoptera exigua* degradating sex pheromones and plant volatile esters. Pestic. Biochem. Phys. 163, 227–234. 10.1016/j.pestbp.2019.11.022 31973861

[B15] Heidari R. Devonshire A. L. Campbell B. E. Dorrian S. J. Oakeshott J. G. Russell R. J. (2005). Hydrolysis of pyrethroids by carboxylesterases from *Lucilia cuprina* and *Drosophila melanogaster* with active sites modified by *in vitro* mutagenesis. Insect Biochem. Mol. Biol. 35 (6), 597–609. 10.1016/j.ibmb.2005.02.018 15857765

[B16] Ishida Y. Leal W. S. (2008). Chiral discrimination of the Japanese beetle sex pheromone and a behavioral antagonist by a pheromone-degrading enzyme. P. Natl. Acad. Sci. U. S. A. 105 (26), 9076–9080. 10.1073/pnas.0802610105 PMC2440356 18579770

[B17] Ishida Y. Leal W. S. (2005). Rapid inactivation of a moth pheromone. P. Natl. Acad. Sci. U. S. A. 102 (39), 14075–14079. 10.1073/pnas.0505340102 PMC1216831 16172410

[B18] Jackson C. J. Liu J. W. Carr P. D. Younus F. Coppin C. Meirelles T. (2013). Structure and function of an insect α-carboxylesterase (αEsterase7) associated with insecticide resistance. P. Natl. Acad. Sci. 110 (25), 10177–10182. 10.1073/pnas.1304097110 PMC3690851 23733941

[B19] Leal W. S. (2013). Odorant reception in insects: Roles of receptors, binding proteins, and degrading enzymes. Annu. Rev. Entomol. 58, 373–391. 10.1146/annurev-ento-120811-153635 23020622

[B20] Levi-Zada A. Byers J. A. (2021). Circadian rhythms of insect pheromone titer, calling, emission, and response: A review. Naturwissenschaften 108 (5), 35. 10.1007/s00114-021-01746-w 34423384

[B21] Li J. Jia Y. Zhang D. Li Z. Zhang S. Liu X. (2023). Molecular identification of carboxylesterase genes and their potential roles in the insecticides susceptibility of *Grapholita molesta* . Insect Mol. Biol. 32 (3), 305–315. 10.1111/imb.12831 36661850

[B22] Li J. Zhang L. (2022). Identification and expression patterns of candidate carboxylesterases in *Carposina sasakii* Matsumura (Lepidoptera: Carposinidae), an important pest of fruit trees. Bull. Entomol. Res. 112 (4), 567–573. 10.1017/S0007485322000244 35670157

[B23] Li R. Zhu B. Hu X.-P. Shi X.-Y. Qi L.-L. Liang P. (2022). Overexpression of PxαE14 contributing to detoxification of multiple insecticides in *Plutella xylostella* (L). J. Agric. Food Chem. 70 (19), 5794–5804. 10.1021/acs.jafc.2c01867 35510781

[B24] Li Y. Sun H. Tian Z. Li Y. Ye X. Li R. (2021). Identification of key residues of carboxylesterase PxEst-6 involved in pyrethroid metabolism in *Plutella xylostella* (L). J. Hazard. Mater. 407, 124612. 10.1016/j.jhazmat.2020.124612 33338816

[B25] Li Z. Q. Cai X. M. Luo Z. X. Bian L. Xin Z. J. Liu Y. (2019). Geographical distribution of *Ectropis grisescens* (Lepidoptera: Geometridae) and *Ectropis obliqua* in China and description of an efficient identification method. J. Econ. Entomol. 112 (1), 277–283. 10.1093/jee/toy358 30508146

[B26] Livak K. J. Schmittgen T. D. (2001). Analysis of relative gene expression data using real-time quantitative PCR and the 2(-Delta Delta C(T)) Method. Method Methods (San Diego, Calif. 25 (4), 402–408. 10.1006/meth.2001.1262 11846609

[B27] Luo Z. X. Li Z. Q. Cai X. M. Bian L. Chen Z. M. (2017). Evidence of premating isolation between two sibling moths: *Ectropis grisescens* and *Ectropis obliqua* (Lepidoptera: Geometridae). J. Econ. Entomol. 110 (6), 2364–2370. 10.1093/jee/tox216 29029204

[B28] Mdeni N. L. Adeniji A. O. Okoh A. I. Okoh O. O. (2022). Analytical evaluation of carbamate and organophosphate pesticides in human and environmental matrices: A review. Molecules 27 (3), 618. 10.3390/molecules27030618 35163876 PMC8840499

[B29] Oakeshott J. G. Claudianos C. Campbell P. M. Newcomb R. D. Russell R. (2005). “Biochemical genetics and genomics of insect esterases,” in Comprehensive molecular insect science. Editors Gilbert L. I. Iatrou K. Gill S. S. (London: Elsevier), 309–361.

[B30] Oakeshott J. G. Claudianos C. Russell R. J. Robin G. C. (1999). Carboxyl/cholinesterases: A case study of the evolution of a successful multigene family. BioEssays news Rev. Mol. Cell. Dev. Biol. 21 (12), 1031–1042. 10.1002/(SICI)1521-1878(199912)22:1<1031:AID-BIES7>3.0.CO;2-J 10580988

[B31] Pfarr K. M. Heider U. Schmetz C. Büttner D. W. Hoerauf A. (2008). The mitochondrial heat shock protein 60 (HSP60) is up-regulated in *Onchocerca volvulus* after the depletion of *Wolbachia* . Parasitology 135 (4), 529–538. 10.1017/s003118200700409x 18205985

[B32] Richmond R. C. Gilbert D. G. Sheehan K. B. Gromko M. H. Butterworth F. M. (1980). Esterase 6 and reproduction in *Drosophila melanogaster* . Science 207 (4438), 1483–1485. 10.1126/science.6767273 6767273

[B33] Shi Y. Li W. Zhou Y. Liao X. Shi L. (2022). Contribution of multiple overexpressed carboxylesterase genes to indoxacarb resistance in *Spodoptera litura* . Pest Manag. Sci. 78 (5), 1903–1914. 10.1002/ps.6808 35066991

[B34] Sun L. Wang Q. Wang Q. Zhang Y. Tang M. Guo H. (2017). Identification and expression patterns of putative diversified carboxylesterases in the tea geometrid *Ectropis obliqua* Prout. Front. Physiol. 8, 1085. 10.3389/fphys.2017.01085 29326608 PMC5741679

[B35] Sun Z. Liu Y. Xu H. Yan C. (2022). Genome-wide identification of P450 genes in *Chironomid Propsilocerus akamusi* reveals candidate genes involved in gut microbiota-mediated detoxification of Chlorpyrifos. Insects 13 (9), 765. 10.3390/insects13090765 36135466 PMC9504602

[B36] Tamura K. Stecher G. Kumar S. (2021). MEGA11: Molecular evolutionary genetics analysis version 11. Mol. Biol. Evol. 38 (7), 3022–3027. 10.1093/molbev/msab120 33892491 PMC8233496

[B37] Vogt R. G. Riddiford L. M. (1981). Pheromone binding and inactivation by moth antennae. Nature 293 (5828), 161–163. 10.1038/293161a0 18074618

[B38] Wang L. L. Huang Y. Lu X. P. Jiang X. Z. Smagghe G. Feng Z. J. (2015). Overexpression of two α-esterase genes mediates metabolic resistance to malathion in the oriental fruit fly, *Bactrocera dorsalis* (Hendel). Insect Mol. Biol. 24 (4), 467–479. 10.1111/imb.12173 25940547

[B39] Wang M. M. Long G. J. Guo H. Liu X. Z. Wang H. Dewer Y. (2021). Two carboxylesterase genes in *Plutella xylostella* associated with sex pheromones and plant volatiles degradation. Pest Manag. Sci. 77 (6), 2737–2746. 10.1002/ps.6302 33527628

[B40] Wang Z. Li H. Zhou X. Tang M. Sun L. Zhan S. (2020). Comparative characterization of microbiota between the sibling species of tea geometrid moth *Ectropis obliqua* Prout and *E. grisescens* Warren. Bull. Entomol. Res. 110 (6), 684–693. 10.1017/S0007485320000164 32741378

[B41] Wei P. Zeng X. Han H. Yang Y. Zhang Y. He L. (2023). Alternative splicing of a carboxyl/choline esterase gene enhances the fenpropathrin tolerance of *Tetranychus cinnabarinus* . Insect Sci. 10.1111/1744-7917.13166 36544383

[B42] Xie M. Ren N. N. You Y. C. Chen W. J. Song Q. S. You M. S. (2017). Molecular characterisation of two α-esterase genes involving chlorpyrifos detoxification in the diamondback moth, *Plutella xylostella* . Pest Manag. Sci. 73 (6), 1204–1212. 10.1002/ps.4445 27717121

[B43] Xu H. Turlings T. C. J. (2018). Plant volatiles as mate-finding cues for insects. Trends Plant Sci. 23 (2), 100–111. 10.1016/j.tplants.2017.11.004 29229187

[B44] Yan Y. Li Y. Wang Q. Tang M. Guo H. Li H. (2022). The expression profiles of chemosensory protein 8 orthologs in two closely related tea geometrid species, *Ectropis obliqua* Prout and *Ectropis grisescens* Warren. J. Tea Sci. 42 (2), 200–210. 10.13305/j.cnki.jts.2022.02.007

[B45] Yan Y. Zhang Y. Tu X. Wang Q. Li Y. Li H. (2020). Functional characterization of a binding protein for Type-II sex pheromones in the tea geometrid moth *Ectropis obliqua* Prout. Pestic. Biochem. Phys. 165, 104542. 10.1016/j.pestbp.2020.02.008 32359552

[B46] Ye J. Mang D. Kang K. Chen C. Zhang X. Tang Y. (2021). Putative carboxylesterase gene identification and their expression patterns in *Hyphantria cunea* (Drury). PeerJ 9, e10919. 10.7717/peerj.10919 33717687 PMC7934681

[B47] Zhang G. H. Yuan Z. J. Zhang C. X. Yin K. S. Tang M. J. Guo H. W. (2014). Detecting deep divergence in seventeen populations of tea geometrid (*Ectropis obliqua* Prout) in China by COI mtDNA and cross-breeding. PLoS One 9 (6), e99373. 10.1371/journal.pone.0099373 24915522 PMC4051655

[B48] Zhang Y.-N. Li J.-B. He P. Sun L. Li Z.-Q. Fang L.-P. (2016). Molecular identification and expression patterns of carboxylesterase genes based on transcriptome analysis of the common cutworm, *Spodoptera litura* (Lepidoptera: Noctuidae). J. Asia-Pac. Entomol. 19 (4), 989–994. 10.1016/j.aspen.2016.07.020

[B49] Zhou X. G. Fu J. Y. Liu S. A. Mao T. F. Xiao Q. Chen X. X. (2016). Molecular detection and sequence analysis of *Wolbachia* strains in *Ectropis obliqua* and *Ectropis grisescens* (Lepidoptera: Geometridae). Chin. J. Appl. Entomol. 53 (4), 782–792. 10.7679/j.issn.2095-1353.2016.097

