# Supplementary material for: Comparative expression profiles of carboxylesterase orthologous CXE14 in two closely related tea geometrid species, Ectropis obliqua Prout and Ectropis grisescens Warren
Source: Front Physiol. 2023 May 24;14:1194997. doi: 10.3389/fphys.2023.1194997 (PMC10244532; doi:10.3389/fphys.2023.1194997)
Supplement: Supplementary file 1 [file DataSheet1.docx]

Comparative expression profiles of carboxylesterase orthologous *CXE14* in two closely related tea geometrid species, *Ectropis obliqua* Prout and *Ectropis grisescens* Warren

Fengshui Yang^1^, Yujie Li^1, 3^, Mengyuan Gao^1, 3^, Qing Xia^1^, Qian Wang^2, *^, Meijun Tang^1^, Xiaogui Zhou^1^, Huawei Guo^1^, Qiang Xiao^1, *^, Liang Sun^1, *^

1. Key Laboratory of Tea Quality and Safety Control, Ministry of Agriculture and Rural Affairs, Tea Research Institute, Chinese Academy of Agricultural Sciences, Hangzhou 310008, China

2. College of Advanced Agricultural Sciences, Zhejiang A & F University, Hangzhou 311300, China

3. Department of Plant Protection, Henan Institute of Science and Technology, Xinxiang 453003, China;

* Correspondence: sunliang01@caas.cn (Liang Sun); wangqianmay@163.com (Qian Wang);

xqtea@mail.tricaas.com (Qiang Xiao)


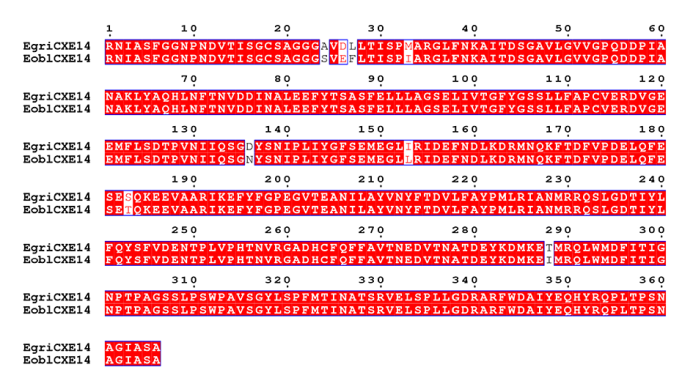


**Figure S1.** The sequence alignment of the two orthologs, EgriCXE14 and EoblCXE14.


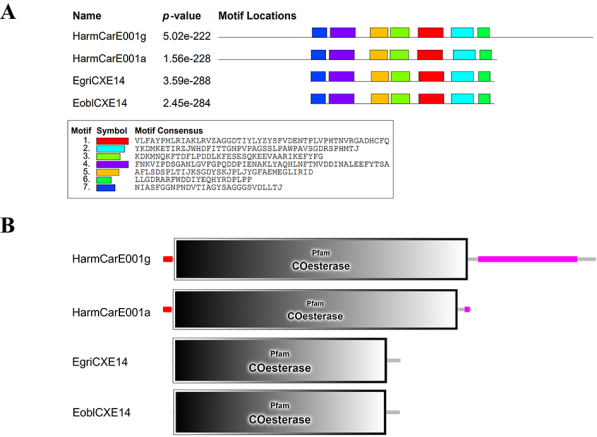


**Figure S2**. The conserved motif (A) and COesterase domain (B) analyses of EgriCXE14 and EoblCXE14 with HarmCarE001g and HarmCarE001a.


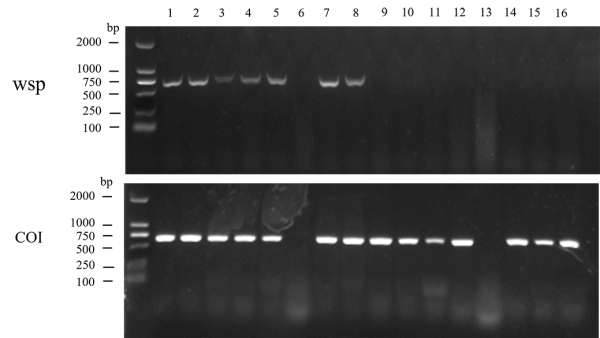


**Figure S3.** The molecular detection of *Wolbachia* removal from *E. grisescens* mutant. The wsp gene was used to examine *Wolbachia* and *COI* gene was used to check the quality of extracted DNA templates. Line1-8 represented the amplification of wsp and *COI* gene from wild *E. grisescens* larvae, line 9-16 represented the amplification of wsp and *COI* gene from mutant *E. grisescens* larvae, in which line 6 and 13 indicated the null that DNA templates was failed to be extracted.

Table S1. Primers used in this study

| **Primer name** | **Sequence (5'-3')** |
| --- | --- |
| *EgriCXE14*-clone-F | AGAAACATCGCTAGTTTTGGGGGAAATCC |
| *EgriCXE14*- clone-R | CGCCGAGGCTATTCCCGCGTT |
| *EoblCXE14*-qPCR-F | ACCAACGAAGATGTAACCAATG |
| *EoblCXE14*-qPCR-R | CAAGACGGCAGAGACGAA |
| *EgriCXE14*-qPCR-F | CTGCGAGGATAAAGGAGT |
| *EgriCXE14*-qPCR-R | CATTGGATAAGCGAAGAGT |

Table S2 Insect CXEs used in phylogenetic tree construction

| **CXEs name** | **Species** | **Accession number** |
| --- | --- | --- |
| EoblCXE14 | *Ectropis obliqua* | ARM65385.1 |
| SexiCXE14 | *Spodoptera exigua* | AEJ38205.1 |
| SexiCXE13 | *Spodoptera exigua* | ADR64701.1 |
| SlitCXE7 | *Spodoptera littoralis* | ACV60234.2 |
| PjapPDE | *Popillia japonica* | AAX58713.1 |
| ApolPDE1 | *Antheraea polyphemus* | AAX58711.1 |
| HarmCarE001a | *Helicoverpa armigera* | ADJ96631.1 |
| HarmCarE001g | *Helicoverpa armigera* | AMO44418.1 |
| BmorCarE | *Bombyx mori* | ACI42854.1 |
| BmanCarE | *Bombyx mandarina* | ABY57297.1 |
| Agamae1A | *Anopheles gambiae* | EAA01826.2 |
| Agamae1F | *Anopheles gambiae* | XP_316296.3 |
| Agambe2C | *Anopheles gambiae* | XP_315381.4 |
| Agambe3C | *Anopheles gambiae* | XP_556011.2 |
| LmigCesA4 | *Locusta migratoria* | AGT95755.1 |
| LmigCesA5 | *Locusta migratoria* | AGT95756.1 |
| DmelEst1 | *Drosophila melanogaster* | AAF54002.3 |
| DmelE7 | *Drosophila melanogaster* | AAF54010.1 |
| DmelEstP | *Drosophila melanogaster* | AAF49945.1 |
| DmelNRT | *Drosophila melanogaster* | CAA37831.1 |
| BdorCarE4 | *Bactrocera dorsalis* | AKN90082.1 |
| BdorCarE6 | *Bactrocera dorsalis* | AKN90083.1 |
| LcaE7 | *Lucilia cuprina* | 4FNG_A |
| MdomE7 | *Musca domestica* | AAD29685.1 |
| NlugCarE | *Nilaparvata lugens* | ACI42853.1 |
| NlugEST1 | *Nilaparvata lugens* | AAG40239.1 |
| LuciNRT | *Lucilia cuprina* | XP_023293592.1 |
| AgamNRT9o | *Anopheles gambiae* | XP_315662.4 |
